# Bacterial arthritis of native joints can be successfully managed with needle arthroscopy

**DOI:** 10.1186/s40634-021-00384-5

**Published:** 2021-08-24

**Authors:** Tobias Stornebrink, Stein J. Janssen, Arthur J. Kievit, Nathaniel P. Mercer, John. G. Kennedy, Sjoerd A. S. Stufkens, Gino M. M. J. Kerkhoffs

**Affiliations:** 1 Department of Orthopedic Surgery, Amsterdam UMC, University of Amsterdam, Amsterdam Movement Sciences, Meibergdreef 9, 1105 AZ Amsterdam, The Netherlands; 2 Academic Center for Evidence Based Sports Medicine (ACES), Amsterdam, The Netherlands; 3 Amsterdam Collaboration for Health and Safety in Sports (ACHSS), International Olympic Committee (IOC) Research Center Amsterdam UMC, Amsterdam, The Netherlands; 4 Department of Orthopaedic Surgery, NYU Langone Health, New York, NY USA

**Keywords:** NanoScope, Needle arthroscopy, Bacterial arthritis

## Abstract

**Purpose:**

To assess the feasibility of needle arthroscopy for management of suspected bacterial arthritis in native joints.

**Methods:**

During a pilot period, patients presenting with symptoms suggestive of native joint bacterial arthritis were eligible for initial management with needle arthroscopy. Procedures were performed in the operating theatre or at the patient bedside in the emergency department or inpatient ward. As our primary outcome measure, it was assessed whether needle arthroscopic lavage resulted in a clear joint. In addition, the need for conversion to standard arthroscopy or arthrotomy, the need for conversion from local to general anaesthesia, complications and the need for additional surgical intervention at follow-up during admission were recorded.

**Results:**

Eleven joints in 10 patients (four males, age range 35 – 77) were managed with needle arthroscopy. Needle arthroscopic lavage resulted in a clear joint in all cases. Conversion to standard arthroscopy or arthrotomy was not needed. Seven procedures were performed at the patient bedside using local anaesthesia. These procedures were well tolerated and conversion to general or spinal anaesthesia was not required. There were no procedure complications. One patient received multiple needle arthroscopic lavages. No further surgical interventions beside the initial needle arthroscopic lavage were required for successful management in other cases.

**Conclusions:**

Needle arthroscopy can be a feasible tool in the initial management of complaints suggestive for native joint bacterial arthritis, providing an effective, quick and well-tolerable intervention in the operating theatre or at the patient bedside, with the potential to relief health systems from need for scarce operating theatre time.

## Background

A suspected bacterial infection of a native joint – so called septic or bacterial arthritis – requires urgent management to control potential life-threatening sepsis and reduce the risk of cartilage destruction. Initial management of a suspected bacterial arthritis consists of joint drainage, followed by antibiotic therapy [7]. Several options are commonly used to drain a bacterially infected joint, ranging from (repeated) needle aspiration to open surgical synovectomy and lavage. Standard arthroscopy is commonly used in daily practice in Europe, allowing for diagnostic joint inspection and therapeutic lavage. However, arthroscopy requires anaesthesia and an operating room, potentially causing diagnostic and treatment delay as well as anaesthesia-related complications and posing a logistic burden on the healthcare system.

Recent technical innovation offers the possibility of 1.9-mm diameter arthroscopy with a disposable and portable arthroscope [13]. This needle arthroscopy uses a tablet for image processing whilst the needle arthroscope can be connected to syringes for distention and rinsing of the joint. Hence, only limited tools are needed (no arthroscopy tower), allowing it to be taken out of the operating theatre and moved around the hospital as a hand-held system. In addition, only small (2.3-mm) portals are required, which are expected to be acceptable for the patient under local anaesthesia [11–13].

These above-mentioned potential benefits of needle arthroscopy might make it very suitable for the (initial) management of suspected native joint bacterial arthritis. Without the need for an operating theatre, the procedure can be performed as a bedside procedure in an office procedure room, emergency department (ER) or inpatient ward. This would expedite diagnosis, treatment, and potentially patient recovery. This possible use and these advantages of needle arthroscopy have not yet been demonstrated – as far as we know. Therefore, this pilot study evaluates first clinical experience with needle arthroscopy for (initial) management of suspected native joint bacterial arthritis.

## Methods

This observational pilot study was conducted in agreement with the 1964 Helsinki Declaration and its later amendments and falls under approval by the Medical Ethics Committee of the University of Amsterdam, with reference number MEC 08/326. In a pilot period between January 2020 and December 2020, all adult (18 years of age or older) patients presenting to the Orthopedic Surgery department of our university hospital (Amsterdam UMC, Amsterdam, The Netherlands) with a clinical suspicion of bacterial arthritis of a native joint were eligible for initial management using needle arthroscopy. The pilot did not alter our standard management of suspected bacterial arthritis, except for the lavage being performed with a needle arthroscope under local portal anaesthesia, instead of a regular arthroscope under general or loco-regional anaesthesia. Whether needle arthroscopy was used instead of standard arthroscopy was left to the discretion of the orthopedic surgeon on call. Patients with a periprosthetic joint infection or a foreign body (e.g. surgical screw or suture anchor) in the affected joint were not eligible for inclusion.

### Needle arthroscope

A 1.9-mm diameter, disposable arthroscope was used for all procedures (NanoScope, Arthrex, Naples, FL). The camera uses an optic chip at the distal end of its camera tube instead of an inner series of conventional rod-lenses. This chip-on-tip technology allows for a semi-rigid frame that remains durable at a diameter of 1.9 mm. In combination with a tailored cannula, the total outer diameter of the arthroscopy portal measures 2.3 mm. Imaging is processed by a tablet-like control unit. Arthroscopic instruments, including a 2-mm diameter probe (NanoProbe, Arthrex, Naples, FL), a 2-mm diameter biter (NanoBiter, Arthrex, Naples, FL), a 2-mm diameter grasper (NanoGrasper, Arthrex, Naples, FL) and a shaver (blades of 2 to 3-mm diameter) are available, allowing for therapeutic interventions during needle arthroscopy.

### Procedure

**Fig. 1:**
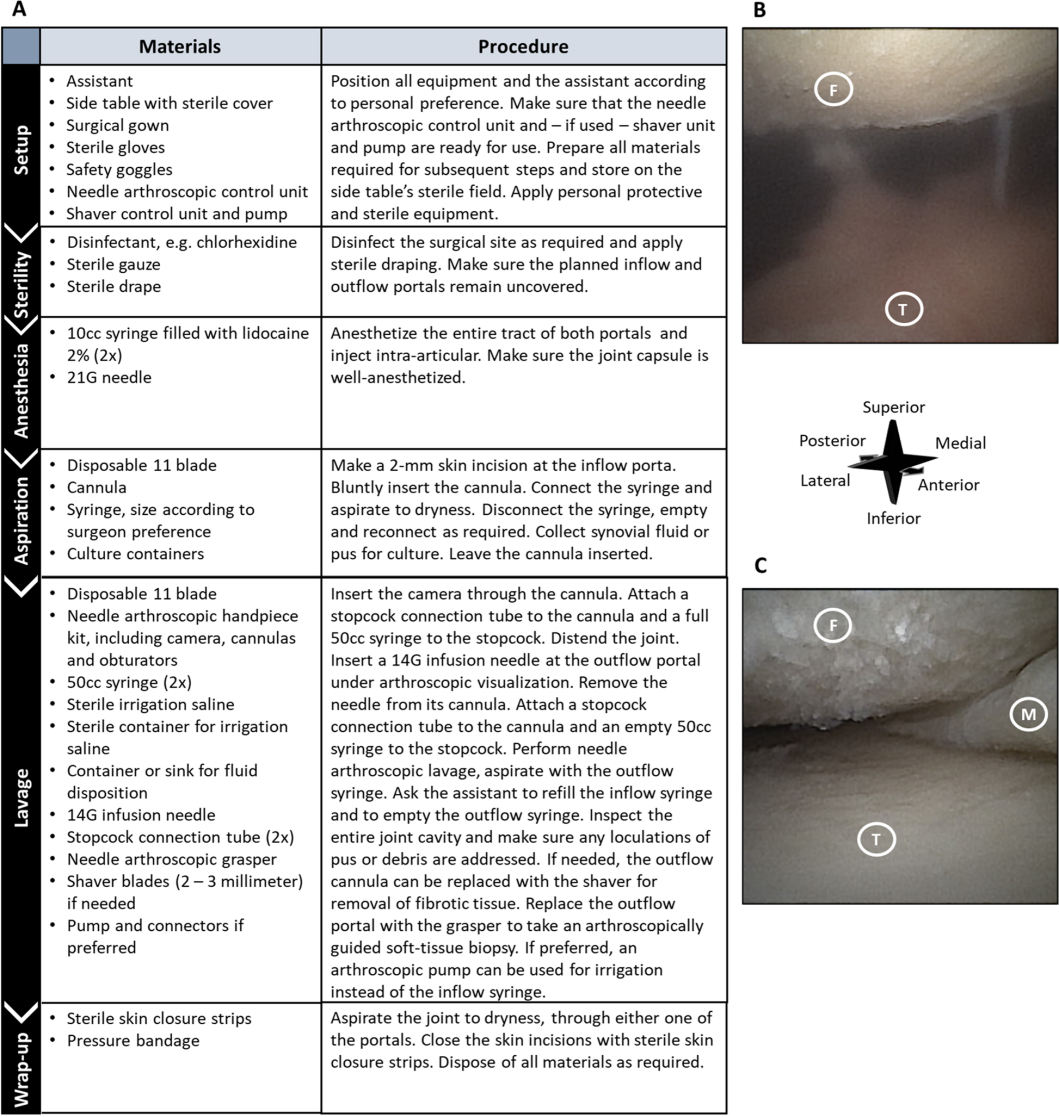
With a stepwise approach (**A**), needle arthroscopic lavage of a suspected native joint bacterial arthritis (**B**) results in a clear joint (**C**). Local anaesthesia is described, yet all other steps equally apply to general or regional anaesthetic procedures. Intra-articular images **B** (before lavage) and **C** (after lavage) were taken from the same anterolateral portal in the same right knee. F indicates the femur, T indicates the tibia, M indicates the meniscus

Procedures were performed in the operating theatre or at the patient bedside in the emergency department or inpatient ward. Figure 1 explains all steps in detail, including materials used in each step. Sterility measures included local skin disinfection, surgical draping and sterile gloves. In case the procedure was performed in the operating theatre, standard general or regional anaesthesia was administered. At the patient bedside, local anaesthesia was used. In case of local anaesthesia, 10 cc lidocaine 2% was injected along the entire tract of planned portals and into the joint cavity. Two portals were used – one for inflow and arthroscope introduction and one for outflow. They were created at standard, joint specific locations. After a 2-mm skin incision and blunt introduction of the cannula, intra-articular fluid or pus was aspirated through the cannula and sent for culture. Subsequently, the needle arthroscope was inserted. A 50 cc syringe was connected to the cannula, and saline was used for distention and lavage. The outflow portal was created under intra-articular visualization and consisted of a large-bore (14G) IV-infusion needle. At the end of the procedure, the incisions were closed with sterile wound closure strips and a bandage, and empirical antibiotic treatment was started.

### Outcome measures

We noted whether needle arthroscopic lavage was deemed successful, and defined this as: successful lavage until macroscopically clear saline and no remaining loculations of pus present upon careful inspection of the joint. In addition, we noted the need for conversion from local anaesthesia to general or regional anaesthesia, the need for conversion from needle arthroscopy to standard arthroscopy or open surgery and occurrence of complications (neurovascular damage, bleeding requiring additional surgery, device failure). Patient follow-up included their entire admission until discharge. During follow-up, it was recorded whether any additional surgical interventions (including needle aspiration, needle arthroscopy, traditional arthroscopy and arthrotomy) were needed to ensure patient recovery.

## Results

Eleven joints in 10 patients were suspected of a bacterial infection and treated using needle arthroscopy upon presentation. Patient age ranged from 35 to 77 years; four patients were male (Table 1). Needle arthroscopy led to successful lavage in all cases (Fig. 1), without a need for conversion to standard arthroscopy or arthrotomy. Eight initial procedures were performed under local anaesthesia in the ER (three cases) and inpatient ward (five cases). Local anaesthesia resulted in a tolerable procedure for all these patient and conversion to regional or general anaesthesia was not required. No procedure related complications occurred. One patient died during admission due to a massive intra-cranial bleeding during chemical thrombolysis on account of critical limb ischemia, for which the patient was initially admitted. Death occurred four days after the needle arthroscopic intervention, and complaints of the affected joint had improved. Synovial fluid cultured positive for a micro-organism in three cases (Table 2). Cultures remained negative in remaining patients. All patients were admitted for intra-venous antibiotic therapy. One patient required two repeated needle arthroscopic lavages due to persistent pain. These additional procedures were both performed in the OR-recovery room, with a single shot femoral block as only anaesthetic. Surgical intervention in addition to needle arthroscopy was not required in remaining cases and all patients were successfully discharged.

**Table 1:** Patient characteristics

**Case**	**Age at presentation**	**Gender**	**Comorbidities**	**Affected joint**	**Clinical features at presentation**	**Lab at presentation**
1	65	Male	Gout, seronegative spondyloarthritis, hypertension	Wrist	T: 35.7	L: 13.0
					Local joint: pain, function loss, swelling, warmth, redness	CRP: 36
2	69	Female	COPD	Knee	T: 36.8	L: 14.6
					Local joint: pain, function loss, swelling, warmth	CRP: 226
3	69	Female	COPD	Knee	T: 37.4	L: 14.6
					Local joint: pain, function loss, swelling, warmth	CRP: 301
4	35	Male	Kidney failure, hypertension, 26 days after conservatively treated ipsilateral patellar fracture	Knee	T: 36.6	L: 8.7
					Local joint: pain, function loss, swelling, warmth	CRP: 169
5	77	Female	Rheumatoid arthritis, diabetes type 2	Ankle	T: 37.6	L: 12.0
					Local joint: pain, function loss, swelling, warmth	CRP: 81
6	66	Male	Hypertension, admitted at presentation on account of infected vascular implant	Knee	T: 37.5	L: 12.9
					Local joint: pain, function loss, swelling	CRP: 137
7	37	Female	Rheumatoid arthritis	Knee	T: 36.7	L: 14.0
					Local joint: pain, function loss, swelling, warmth	CRP: 346
8	68	Female	Rheumatoid arthritis, osteoarthritis, stroke, diabetes type 2, atrial fibrillation, heart failure	Shoulder	T: 38.8	L: 7.9
					Local joint: Pain, loss of function, swelling, warmth	CRP: 161
9	58	Female	Kidney failure, hypertension, Sjogren’s syndrome, gout	Knee	T: 37.0	L: 7.3
					Local joint: pain, function loss, swelling	CRP: 270
10	71	Female	Peripheral arterial occlusive disease, hypertension, atrial fibrillation	Knee	T: 37.0	L: 14.9
					Local joint: pain, loss of function, swelling, warmth, redness	CRP: 183
11	54	Male	Transtibial amputation (Left), hypertension, diabetes type 1	Knee	T: 37.1	L: 25.3
					Local joint: pain, loss of function, swelling, warmth, redness	CRP: 322

*T* denotes temperature in degrees Celsius, *L* denotes the leucocyte count times 10^9^/L, *CRP* denotes the C-reactive protein in mg/L

**Table 2:** Procedure characteristics and outcome

**Case**	**Anaesthesia**	**Procedure location**	**Complications**	**Additional surgery required during admission**	**Micro-organism cultured**
1	General	OR	No	No	No
2	Local	ER	No	No	No
3	Local	Inpatient ward	No	No	No
4	General	OR	No	No	No
5	Local	ER	No	No	No
6	Local	Inpatient ward	No	No	No
7	General	OR	No	No	Streptococcus agalactiae
8	Local	Inpatient ward	No	No	No
9	Local	Inpatient ward	No	No	S. Aureus
10	Local	Inpatient ward	Yes, not related to the suspected bacterial arthritis or needle arthroscopic procedure	No	No
11	Local	ER	No	Yes, two additional needle arthroscopic lavages (11.2 & 11.3)	Streptococcus agalactiae
11.2	Single shot femoral block	Recovery room	No	“	“
11.3	Single shot femoral block	Recovery room	No	“	“

## Discussion

The main finding of this study was that needle arthroscopy could be successfully used as the initial step in the management of a suspected bacterial arthritis of a native joint. Without a need to convert to standard arthroscopy or arthrotomy, needle arthroscopic lavage resulted in a macroscopically clear joint in all cases. Procedures were successfully performed at the patient bedside in the ER and inpatient ward with local administration of lidocaine as the only anaesthetic. There were no complications and there was no need for further surgical intervention or needle aspiration.

Currently, the initial management of suspected bacterial arthritis includes either surgical lavage or repeated arthrocentesis [2]. This pilot study showed the feasibility of needle arthroscopy in reaping the benefits of both approaches. Intra-articular visualization and 2-mm diameter instruments allow for thorough diagnostic inspection of the joint, irrigation with large amounts of fluids, identification and removal of localized collections of pus and debris – as all propagated by proponents of a surgical approach to suspected bacterial arthritis. Yet simultaneously, it is a quick procedure that can be performed at the patient bedside under local anaesthesia, without causing extensive soft-tissue trauma – all advantages known to arthrocentesis as well.

Local anaesthesia with lidocaine was sufficient for a well-tolerable intervention. Needle arthroscopy has been successfully performed under local anaesthesia before [17], and various interventional techniques are emerging – including repair of cruciate ligaments and meniscal tears [4, 14]. Yet, whether more extensive debridement including synovectomy is tolerable for the patient as well, is a subject for future research. Being able to use only local anaesthesia may be beneficial especially in the setting of suspected bacterial arthritis – an event occurring predominantly in children and older adults – as older age, acute surgery and sepsis are all risk factors for anaesthesia-related complications [3, 6, 10].

In the past, a move from the operating theatre to the patient bedside has resulted in effective and safe surgery, with happier patients and at lower costs [15]. Eliminating the need for conventional surgery can have an especially large impact in the acute care setting, where surgical costs form a substantial proportion of total costs of care [5]. In addition, the need for general anaesthesia and an operating theatre may delay treatment due to pre-anaesthetic tests that have to be conducted or a scarcity of resources – with the current COVID-19 pandemic highlighting the relevance of scarcity and even resulting in scoring systems to distribute scarce urgent surgical care [8, 9]. Avoiding treatment delay is especially important in patients with bacterial arthritis, where only prompt intervention can avoid morbidity and even mortality.

Lavage saline was administered through the camera portal. That is, saline travelled between the inner cannula wall (2.2-mm), and the outer camera wall (1.9-mm). A second portal was created for outflow. The diameter of the inflow portal was substantially smaller compared to standard arthroscopy, which decreases the intra-articular passage speed of irrigation saline [16]. Inflow proved to generate sufficient pressure for joint distention similar to standard arthroscopy. Yet, the time that was required to pass a sufficient amount of saline indeed seemed longer compared standard arthroscopy. Nonetheless, evidence suggests that the rinsing pressure that can be generated with syringes is sufficient for adequate lavage of a bacterially infected surface [1]. In an in-vitro setting, small-bore syringe irrigation of bacterially infected biological surfaces – including cancellous bone – provided effective reduction of the bacterial load, statistically equal to a large-bore jet lavage system [1]. In our pilot patients, lavage resulted in a clear joint in all cases, without a need for further surgical intervention at a later stage during admission. This underscores the feasibility of bedside needle arthroscopy as a minimally invasive, quick and effective first intervention in acute arthritis. Nevertheless, we do recommend to consider that needle arthroscopic lavage may have been inferior compared to what we are used to from standard arthroscopy in case of a non-recovering patient.

This pilot study should be interpreted in light of its limitations. Rather than providing proof of effective treatment, the limited number of patients merely indicates the feasibility of needle arthroscopy in the initial management of suspected native joint bacterial arthritis. Only knee, wrist, ankle and shoulder cases were treated so far. Needle arthroscopic treatment of other joints as the hip may be more difficult. A microorganism was cultured in the synovial fluid in only three out of 11 cases (27%). A negative culture alone cannot rule out bacterial arthritis in case of high clinical suspicion [2], which applied to all included patients. Nevertheless, the results of this pilot study should be transferred to culture-proven cases with care until examined in a sufficiently powered sequel study.

**Fig. 2:**
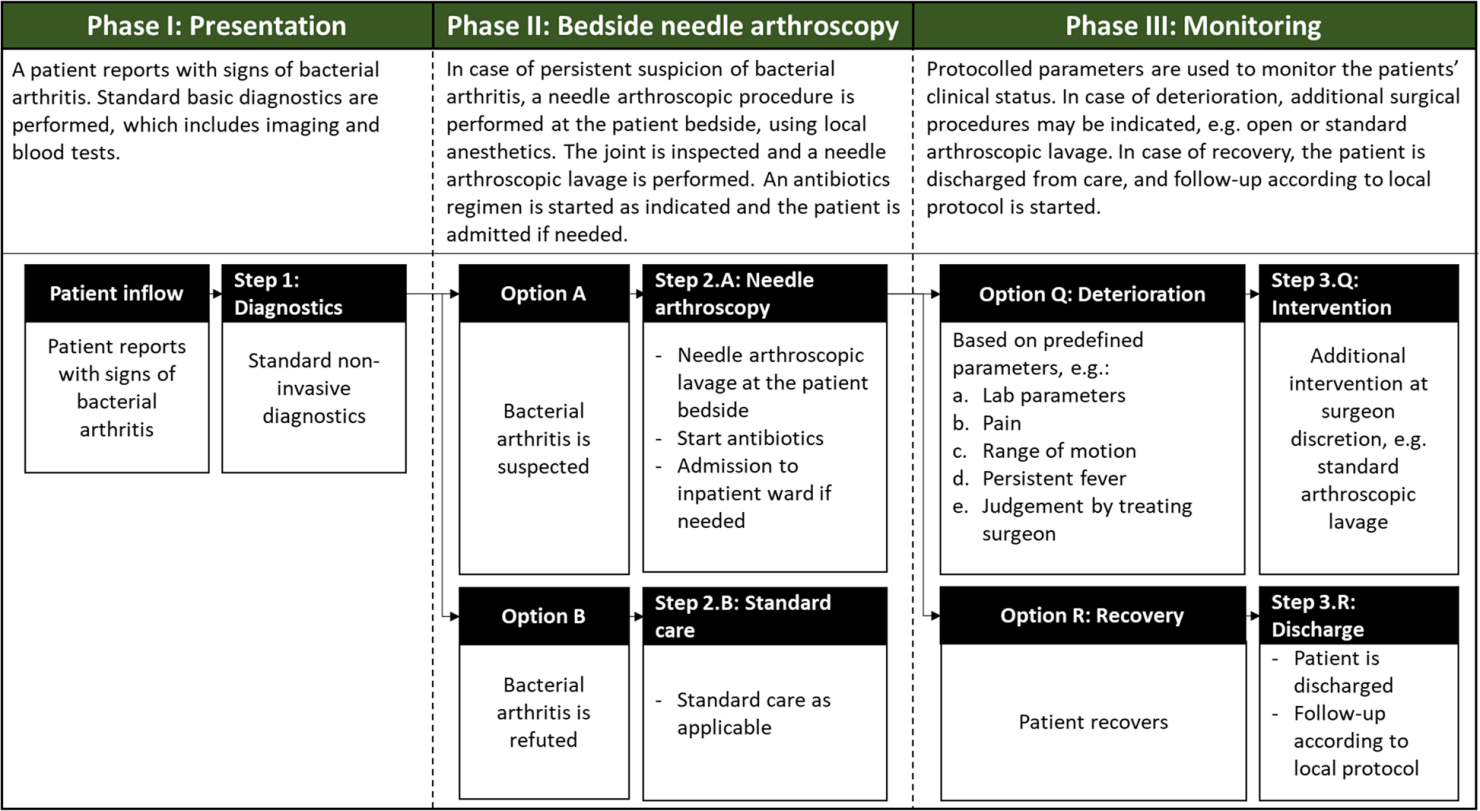
Needle arthroscopy can successfully be used as the initial intervention in suspected bacterial arthritis of a native joint. Incorporating needle arthroscopy in the management protocol may result in less use of surgical interventions as standard arthroscopy or open surgery, whilst standard surgery can always be deployed as a back-up intervention for non-improving patients

We suggest using needle arthroscopy as the initial step in the management of suspected bacterial arthritis of a native joint. Figure 2 provides a potential management protocol. Following initial needle arthroscopic lavage, an antibiotic regimen according to local protocol should commence. At surgeon discretion, patients may further be treated with repeated needle arthroscopy or arthrocentesis to provide additional relief and monitor disease activity. In case of disease progression – e.g. a persistent fever, increasing pain, non-improving range of motion or deteriorating lab parameters – more invasive surgery should be considered. A prospective evaluation of this management protocol was started in our university medical centre. This prospective study will further scrutinize the benefit of needle arthroscopy in treating native joint bacterial arthritis.

## Conclusions

This feasibility study indicates that needle arthroscopy could be a successful tool in the initial management of complaints suggestive for native joint bacterial arthritis, providing an effective, quick and well-tolerable intervention in the operating theatre or at the patient bedside. It has the potential to decrease demand for scarce operating theatre time, which can subsequently be more effectively allocated to other indications. Patients unfit for surgery at the time of suspected diagnosis can be treated more promptly.

## Data Availability

The datasets used and/or analysed during the current study are available from the corresponding author on reasonable request.
